# Refining precision prognostics in multiple myeloma: loss of miR-221/222 cluster in CD138+ plasma cells results in short-term progression and worse treatment outcome

**DOI:** 10.1038/s41408-025-01248-2

**Published:** 2025-03-15

**Authors:** Konstantinos Soureas, Panagiotis Malandrakis, Maria-Alexandra Papadimitriou, Christos Minopoulos, Ioannis Ntanasis-Stathopoulos, Christine-Ivy Liacos, Maria Gavriatopoulou, Efstathios Kastritis, Meletios-Athanasios Dimopoulos, Andreas Scorilas, Margaritis Avgeris, Evangelos Terpos

**Affiliations:** 1 Department of Biochemistry and Molecular Biology, Faculty of Biology, National and Kapodistrian University of Athens, Athens, Greece; 2 Laboratory of Clinical Biochemistry—Molecular Diagnostics, Second Department of Pediatrics, School of Medicine, National and Kapodistrian University of Athens, “P. & A. Kyriakou” Children’s Hospital, Athens, Greece; 3 Department of Clinical Therapeutics, School of Medicine, National and Kapodistrian University of Athens, Alexandra General Hospital, Athens, Greece

**Keywords:** Myeloma, Preclinical research, Translational research

## Abstract

The persistence of high relapse rates and therapy resistance continues to challenge the effective management of multiple myeloma (MM). The identification of novel MM-specific molecular markers could ameliorate risk-stratification tools and accurately identify high-risk patients towards personalized prognosis and therapy. miRNA-seq analysis of CD138+ plasma cells (n = 24) unveiled miR-221-3p and miR-222-3p (miR-221/222 cluster) as the most downregulated miRNAs in R-ISS III compared to R-ISS I/II patients. Subsequently, miR-221/222 levels were quantified by RT-qPCR in CD138+ plasma cells of our screening cohort (n = 141), assessing patients’ mortality and disease progression as clinical endpoints. Internal validation was performed by bootstrap analysis, while clinical benefit was estimated by decision curve analysis. Kryukov et al. (n = 149) and Aass et al. (n = 86) served as institutional-independent validation cohorts. Loss of miR-221/222 cluster was strongly associated with patients’ short-term progression and poor overall survival, which was confirmed by Kryukov et al. and Aass et al. validation cohorts. Intriguingly, miR-221/222-fitted multivariate models offered superior risk-stratification within R-ISS staging and risk-based cytogenetics. Moreover, miR-221/222 loss could effectively discriminate optimal 1st-line treatment responders with inferior treatment outcome. Our study identified the loss of miR-221/222 cluster as a powerful independent predictor of patients’ post-treatment progression, ameliorating prognosis and supporting precision medicine in MM.

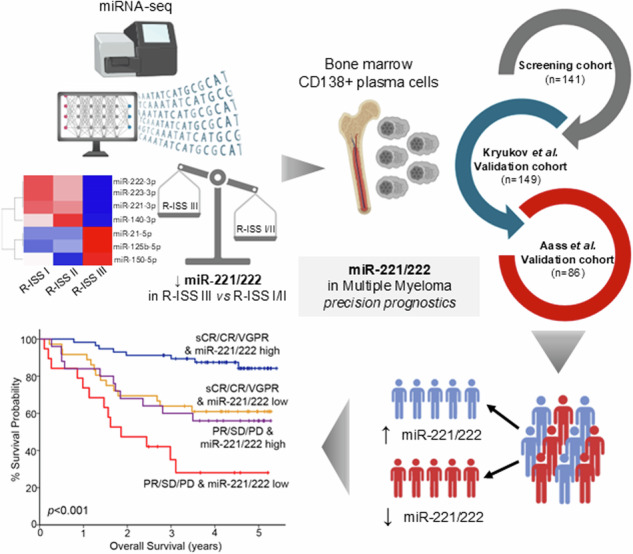

## Introduction

Multiple myeloma (MM) ranks as the second most prevalent hematologic malignancy, comprising roughly 20% of the blood cancers associated mortality in the western world [1, 2]. Typically, MM is preceded by the benign monoclonal gammopathy of undetermined significance (MGUS), which may progress to asymptomatic smoldering myeloma (sMM), before presenting clinical symptoms necessitating treatment [3]. The transition between disease stages and the development of treatment resistance are intertwined with complex pathophysiology, involving DNA damage, epigenetic irregularities, and the implication of bone marrow (BM) microenvironment [4, 5].

Despite the substantial advancements in response rates and survival of the patients, driven by the introduction of novel therapeutic agents over the past two decades, MM remains an incurable malignancy due to the emergence of constant relapses and treatment resistance [3, 4, 6]. Currently, the deployment of Revised International Staging System (R-ISS) represents the main established risk-stratification framework for disease prognosis and treatment decisions in the clinical setting; however, patients within the same stage could present great heterogeneous outcomes, rendering the need of the delineation of novel clinical tools supporting personalized prognosis and tailored therapeutics in MM [7–9].

MicroRNAs (miRNAs; 18–25 nucleotides) have emerged as an essential element of the non-coding transcriptome, playing a crucial role in post-transcriptional gene expression regulation (2024 Nobel Prize in Physiology or Medicine to Ambros V. and Ruvkun G.) [10–12]. The regulatory function of miRNAs predominantly occurs through their binding to the 3′-untranslated region (3′-UTR) of target mRNAs, recruiting the RNA-induced silencing complex (RISC), which subsequently facilitates the inhibition of translation and mRNA decay. A substantial proportion of miRNAs present significant conservation in their “seed region” across species, and more than 60% of protein-coding genes have been verified as targets for multiple miRNAs [12, 13]. This highlights the intricate and profound effects of miRNAs on various physiological and developmental processes, including cell proliferation, quiescence, and apoptosis [14]. In this context, the significant progress in high-throughput technologies in recent years has enabled the identification and development of miRNA prognostic signatures, linking their aberrant expression with various human malignancies, including MM [15–18].

The aim of our present study is to offer a comprehensive insight into miRNA profiles in MM, with the intention of identifying potential R-ISS-associated miRNAs that hold prognostic significance for patients’ tailored management. miRNA-seq analysis of CD138+ plasma cells revealed miR-221-3p and miR-222-3p (miR-221/222) as the most downregulated miRNAs in R-ISS III compared to R-ISS I/II patients, prompting us to further evaluate for the first time the clinical potential of miR-221/222 cluster loss in MM treatment outcome, using a screening MM cohort (n = 141 patients) and two external institutional-independent validation cohorts, the Kryukov et al. (n = 149) [19] and the Aass et al. (n = 86) [20] cohorts. Our findings highlighted that miR-221/222 loss was strongly associated with worse post-treatment survival and contributed to superior clinical benefit in MM prognostication. Notably, multivariate models fitted for miR-221/222 levels offered superior risk-stratification within R-ISS staging and risk-based cytogenetics, enhancing prediction of treatment outcome, while miR-221/222 loss could effectively distinguish optimal 1st-line treatment responders with inferior treatment outcome.

## Methods

### Screening cohort

The screening cohort consisted of 141 newly diagnosed MM patients, non-previously treated, based on the standard criteria of the International Myeloma Working Group (IMWG) [21, 22]. BM aspirates were collected during disease diagnosis at the Department of Clinical Therapeutics, “Alexandra” Hospital, Athens, Greece. At the time of diagnosis, patients were subjected to baseline assessment using blood, biochemical, and imaging tests. Cytogenetic abnormalities were identified using conventional cytogenetic protocols and interphase fluorescence in situ hybridization (FISH) at BM aspirate from trephine biopsy, while bone disease was assessed by whole-body low dose computed tomography (WBLDCT).

**Fig. 1:**
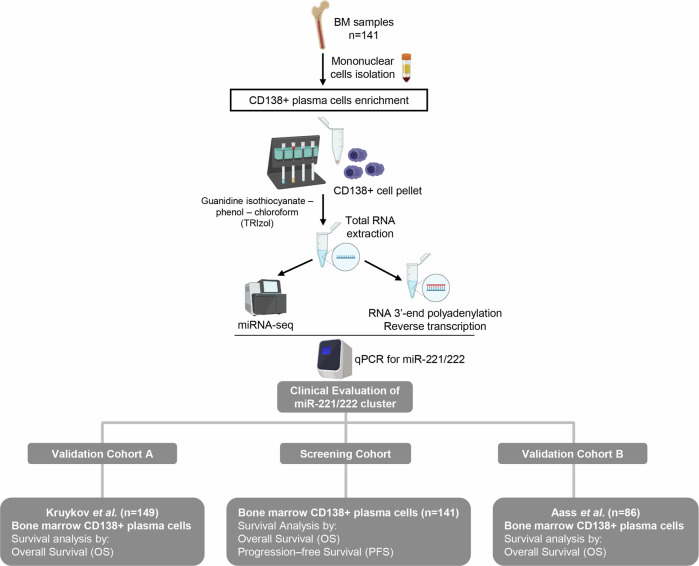
Graphical representation of the study workflow.

Of the 141 recruited patients, 125 (88.7%) patients received bortezomib-based regimens, while 12 (8.5%) of them were treated with lenalidomide-based regimens. Based on R-ISS staging, 22.7%, 53.9%, and 18.4% of the MM patients were classified as R-ISS I, II, and III, respectively, while bone disease was detected in 66.6% of the patients at MM diagnosis. Response to treatment was assessed monthly according to IMWG criteria with blood and urine tests. The study was conducted in accordance with the 1975 Declaration of Helsinki ethical standards, as revised in 2008 and approved by the Ethics Committee of “Alexandra” Hospital, Athens, Greece. Prior to sampling, informed consent was obtained from all participating patients. Patients’ clinicopathological data are summarized in Table 1, while the representation of the study is illustrated in Fig. 1.

**Table 1:** Clinicopathological features of the screening cohort.

Multiple myeloma patients	No. of patients (n = 141)
R-ISS stage	
R-ISS I	**32** (22.7%)
R-ISS II	**76** (53.9%)
R-ISS III	**26** (18.4%)
Missing data	7
ISS Stage	
ISS I	**39** (27.7%)
ISS II	**42** (29.8%)
ISS III	**59** (41.8%)
Missing data	1
Prior sMM/MGUS	
Yes	**20** (14.1%)
No	**121** (85.8%)
Gender	
Male	**79** (56.0%)
Female	**62** (44.0%)
Therapy	
Bortezomib-based regimens	**125** (88.7%)
Lenalidomide-dexamethasone	**12** (8.5%)
Other	2 (1.4%)
Not complete treatment	2 (1.4%)
Bone disease	
Yes	**94** (66.6%)
No	**36** (25.5%)
Missing data	11
HDM/ASCT	
Yes	**40** (28.4%)
No	**101** (71.6%)
B2M	
< 5.5 mg/l	**82** (58.6%)
≥5.5 mg/l	**58** (41.4%)
Missing data	1
LDH	
≤ 220 U/l	**110** (78.0%)
>220 U/l	**31** (22.0%)
Marrow plasma cells	
< 60%	**59** (41.8%)
≥60%	**82** (58.2%)
Response to 1st-line	
sCR, CR, VGPR	**93** (66.0%)
PR, SD, PD	**44** (31.2%)
Missing data	4
Disease monitoring	
Follow-up patients	**141**
Relapse	**55** (39.0%)
Death / Alive	**50** (35.5%) / **91** (64.5%)
Progression / Progression-free survival	**82** (58.2%) / **59** (41.8%)

*HDM/ASCT* high dose melphalan therapy with autologous stem cell transplantation, *sCR* stringent complete response, *CR* complete response, *VGPR* very good partial response, *PR* partial response, *SD* stable disease, *PD* progressed disease.

### Validation cohorts

Kryukov et al. (n = 151) and Aass et al. (n = 86) were used as independent validation cohorts. Kryukov et al. cohort consists of 151 MM patients and transcriptional profiling of CD138+ plasma cells was generated by microarray analysis using the Affymetrix GeneChip Human Gene 1.0 ST Array platform, while complete follow-up data are available for 149 patients [19]. Aass et al. cohort consists of 86 MM patients and transcriptional profiling of CD138+ plasma cells were performed by small RNA-sequencing using NextSeq 500 System from Illumina [20].

### CD138+ plasma cells isolation and RNA extraction

BM aspirates were collected in EDTA tubes, and mononuclear cells were isolated using Ficoll-Paque. CD138+ plasma cells were then enriched through magnetic cell sorting employing immunomagnetic microbeads coated with anti-CD138 monoclonal antibody (MACS CD138 microbeads, Miltenyi-Biotec GmbH, Bergisch Gladbach, Germany). Subsequently, total RNA was extracted using TRI-Reagent (Molecular Research Center, Cincinnati, OH, USA) according to manufacturer’s protocol. RNA was dissolved in RNA Storage Solution (Ambion, Austin, TX, USA) while its concentration was determined using Qubit 2.0 Fluorometer (Invitrogen, Carlsbad, CA, USA).

### miRNA-seq

Next generation sequencing (NGS) libraries were constructed from 8 R-ISS I, 8 R-ISS II, and 8 R-ISS III MM, using the QIAseq miRNA Library Kit (Qiagen, Hilden, Germany) and 500 ng of total RNA from CD138+ plasma cells as starting template. Briefly, adapters were ligated to the 3’- and 5’-ends of miRNAs sequentially, UMI (Unique Molecular Identifier)-based cDNA synthesis and cleanup was performed, followed by amplification with a universal forward primer and indexing reverse primers. The quality assessment of the constructed libraries was carried out in the 2100 Bioanalyzer (Agilent Technologies, Santa Clara, CA, USA), using the Agilent High Sensitivity DNA Kit (Agilent). Finally, miRNA-seq was performed using Illumina NextSeq 550 system (Illumina, San Diego, CA, USA), producing 75 bp single sequencing reads for each miRNA-seq library.

### Bioinformatic analysis

The quality control of the sequenced libraries was accomplished with FastQC software, while adapter trimming was performed utilizing Trim Galore algorithm. Sequencing reads with lengths <16 nt and >40 nt were removed from the datasets used for downstream analysis. Finally, miRDeep2 was used for mapping mature miRNAs [23] (according to miRbase database release 22.1 [24]) on human genome sequence (hg38) and for the elucidation of miRNA profiling across the investigated samples. For each tested sample, to avoid misinterpretations resulting from the higher technical noises of low read counts, only miRNAs candidates with positive miRDeep2 score and >50 unnormalized read counts were used for further analysis.

### Polyadenylation of total RNA and first-strand cDNA synthesis

200 ng of total RNA was polyadenylated at the 3’-end using 1 U of *E. coli* poly(A) polymerase (New England Biolabs Inc., Ipswich, MA, USA) and 800 μM ATP, in a 10 μL reaction, at 37 ^o^C for 60 min, followed by a 65 °C for 10 min enzyme inactivation step. Polyadenylated RNA was used as a template for first-strand cDNA synthesis. The 20 μL reaction consisted of 200 U M-MLV Reverse Transcriptase (Invitrogen), 40 U RNaseOUT Recombinant Ribonuclease Inhibitor (Invitrogen), 10 mM dNTPs Mix, and 250 nM oligo-dT adapter 5′-GCGAGCACAGAATTAATACGACTCACTATAGGTTTTTTTTTTTTVN-3′ (V = G, A, C and N = G, A, T, C). The applied thermal protocol was 37 ^o^C for 60 min and 70 ^o^C for 15 min.

### Quantitative real-time PCR (qPCR)

SYBR Green I dye-based quantitative real-time PCR (qPCR) assays were designed and implemented to measure miR-221/222 levels. Based on published sequences (NCBI RefSeq: NR_029635.1 for miR-221-3p, NR_029636.1 for miR-222-3p and NR_002745.1 for SNORD48), specific forward primers for miR-221-3p (5′‐GCTACATTGTCTGCTGGGTTTCA‐3′), miR-222-3p (5′-GAGCTACATCTGGCTACTGGGTAA-3′), and small nucleolar RNA, C/D box 48 (SNORD48), frequently annotated as RNU48, (5′-TGATGATGACCCCAGGTAACTCT-3′). The specific forward primers and a universal reverse primer (5’-GCGAGCACAGAATTAATACGAC-3’), complementary to the oligo-dT adapter, were used for the amplification of a 63 bp specific amplicon of miR-221 and miR-222, and a 105 bp specific amplicon of RNU48, respectively. The QuantStudio™ 5 Real-Time PCR System (Applied Biosystems) was used for the qPCR assays. The 10 μL reactions were performed including Kapa SYBR® Fast Universal 2X qPCR Master Mix (Kapa Biosystems, Inc., Woburn, MA, USA), 200 nM of each PCR primer, and 1 ng of cDNA template. The thermal protocol was 95 ^o^C for 3 min for polymerase activation, followed by 40 cycles of denaturation at 95 ^o^C for 15 s and primer annealing and extension at 60 ^o^C for 1 min. Melting curve analysis and agarose gel electrophoresis were conducted to verify the specificity of the reaction. To ensure reproducibility, reactions were performed in duplicates. The 2^−ΔΔCT^ relative quantification (RQ) method was employed to analyze the expression levels of miR-221/222, utilizing RNU48 as endogenous reference control for normalization purposes.

### Statistical analysis

IBM SPSS Statistics 20 software (IBM Corp., Armonk, New York, USA) was used for statistical analysis. Survival analysis was conducted assessing Kaplan–Meier curves using log-rank test and Cox proportional regression analysis. X-tile algorithm was applied for the adoption of optimal cut-off values of miR-221/222 levels. Internal validation was performed by bootstrap Cox proportional regression analysis based on 1000 bootstrap samples. Finally, the clinical benefit of miR-221/222 in patients’ prognosis and treatment outcome was evaluated using decision curve analysis (DCA), according to Vickers et al. [25], by STATA 13 software (StataCorp LLC, College Station, TX, USA).

## Results

### miRNA-seq profiling of CD138+ plasma cells

**Fig. 2:**
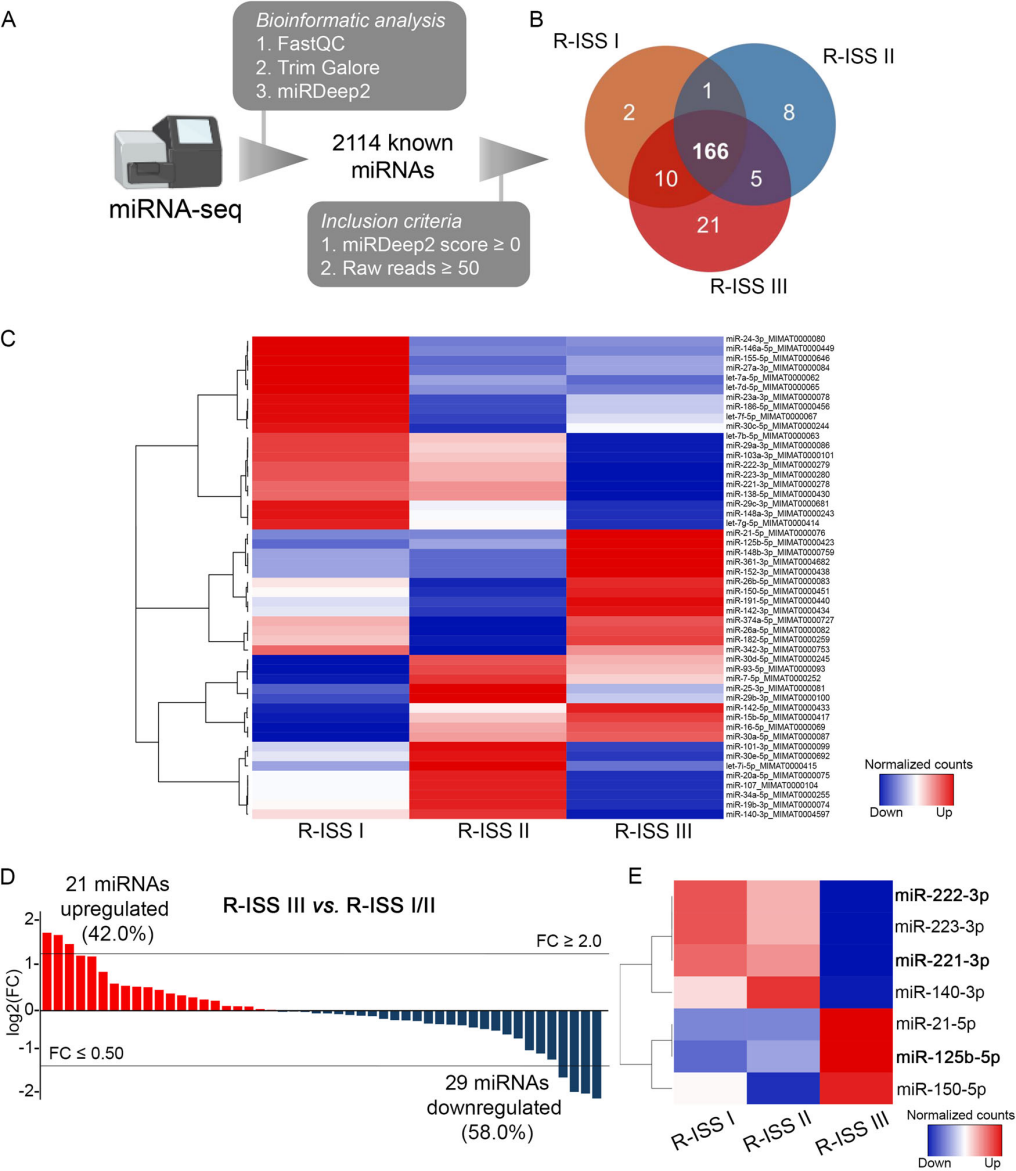
miRNA profiling of CD138+ plasma cells. **A** Experimental workflow of miRNA-seq analysis of CD138+ plasma cells. **B** Venn diagram representing shared miRNAs between R-ISS stages meeting the inclusion criteria. **C** Heatmap of the differently expressed miRNAs between R-ISS stages. **D** Bar plot of miRNAs log2FC in R-ISS III vs R-ISS I/II. **E** Heatmap of the altered miRNAs between R-ISS III and R-ISS I/II with a FC ≥ 2 or FC ≤ 0.5. Color gram depicts high (red) and low (blue) levels of miRNAs. FC fold change.

To investigate miRNAs profile of CD138+ plasma cells, miRNA-seq libraries were constructed from 24 MM patients (8 R-ISS I, 8 R-ISS II, and 8 R-ISS III). The principal workflow is schematically presented in Fig. 2A. More specifically, FastQC algorithm was used for the quality control of the libraries and Trim Galore algorithm for adapter trimming, excluding noise, while miRDeep2 was utilized for mature miRNA mapping; where 2114 known mature miRNAs were successfully mapped on human genome sequence (hg38). To filter-out false-positive signals, we have excluded miRNAs with negative miRDeep2 score, while to avoid the bias from the higher technical noises for low read counts, miRNAs with <50 raw reads were excluded from downstream analysis, narrowing down to 166 mature miRNAs, that were concurrently expressed in all three R-ISS stages (Fig. 2B). Focusing on the top 30% more abundant targets (Fig. 2C), 21 miRNAs were upregulated (42.0%), while 29 were downregulated (58.0%) in R-ISS III *vs*. R-ISS I/II (Fig. 2D, Supplementary Table 1). Among them, 3 and 4 miRNAs presented fold change (FC) ≥ 2 and ≤0.5 (Fig. 2E), respectively. Overall, miR-221-3p and miR-222-3p were the most downregulated miRNAs in R-ISS III compared to R-ISS I/II, while miR-125b-5p was highlighted as the most upregulated one, triggering us to further investigate their clinical impact in MM prognosis and patients’ treatment outcome.

### Loss of miR-221/222 cluster is associated with short-term progression and poor survival outcome

The initial analysis of a sub-cohort of 46 MM patients (~33% of the screening cohort) highlighted the significant association of miR-221/222 (Supplementary Fig. 1A) loss with worse OS of MM patients (Supplementary Fig. 1B), while no statistically significant correlation was documented for miR-125b levels (Supplementary Figure 1C), supporting the complete clinical evaluation of miR-221/222 cluster in MM patients’ outcome.

**Fig. 3:**
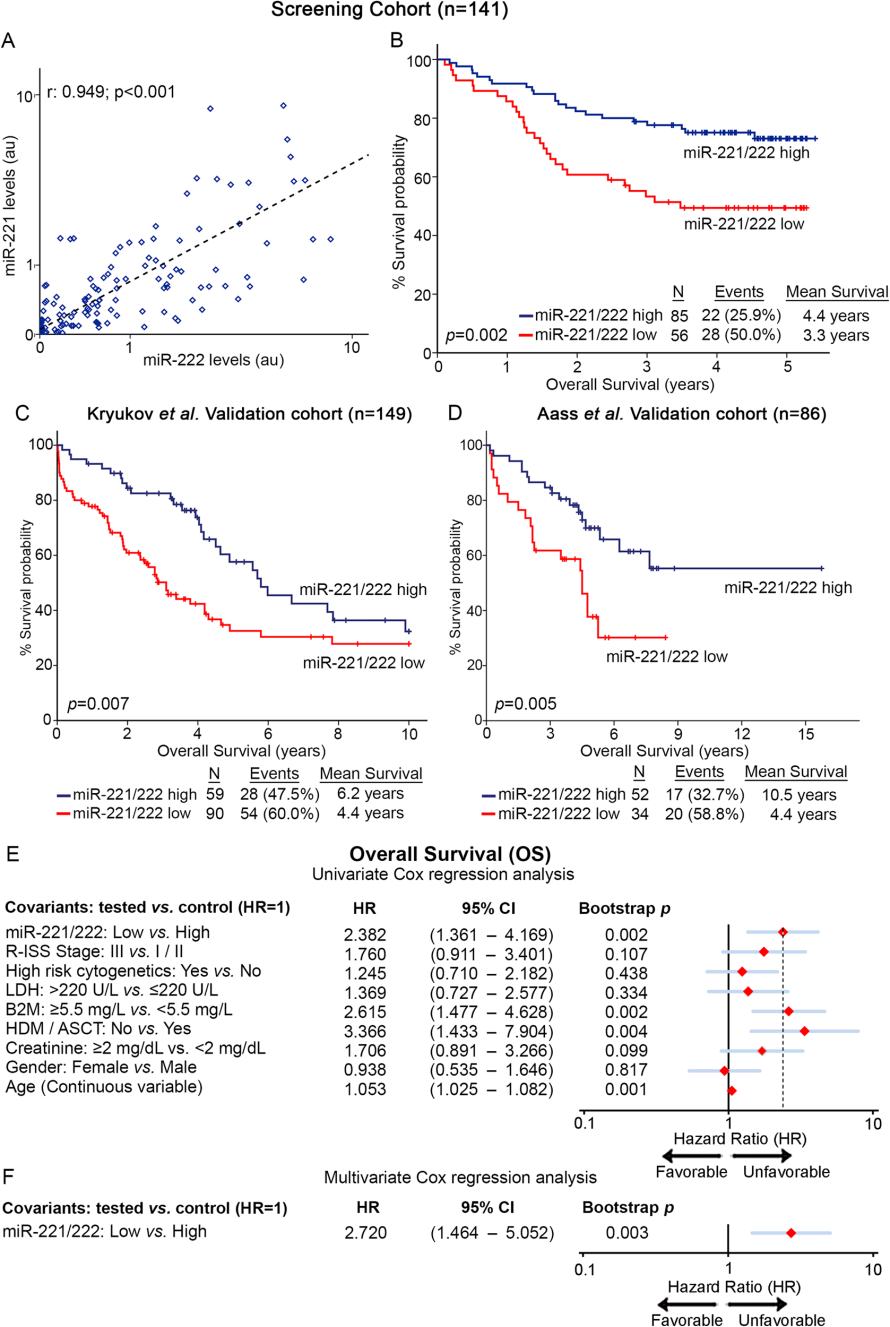
miR-221/222 loss is strongly associated with worse survival outcome following treatment. **A** Pearson correlation of miR-221 and miR-222 levels in the screening cohort. **B**–**D** Kaplan–Meier survival curves for overall survival (OS) of the screening cohort (**B**), as well as of Kruykov et al. (**C**) and Aass et al. (**D**) validation cohorts. *p* values calculated by log-rank test. Forest plots of the univariate (**E**) and multivariate (**F**) Cox regression analysis for the OS of the patients. Multivariate analysis adjusted for miR-221/222 levels, R-ISS stage, high-risk cytogenetics, LDH, B2M, and creatinine levels, HDM/ASCT, gender, and age. Internal validation was performed by bootstrap Cox proportional regression analysis based on 1000 bootstrap samples. HR: Hazard Ratio, 95% CI: 95% confidence interval of the estimated HR.

**Fig. 4:**
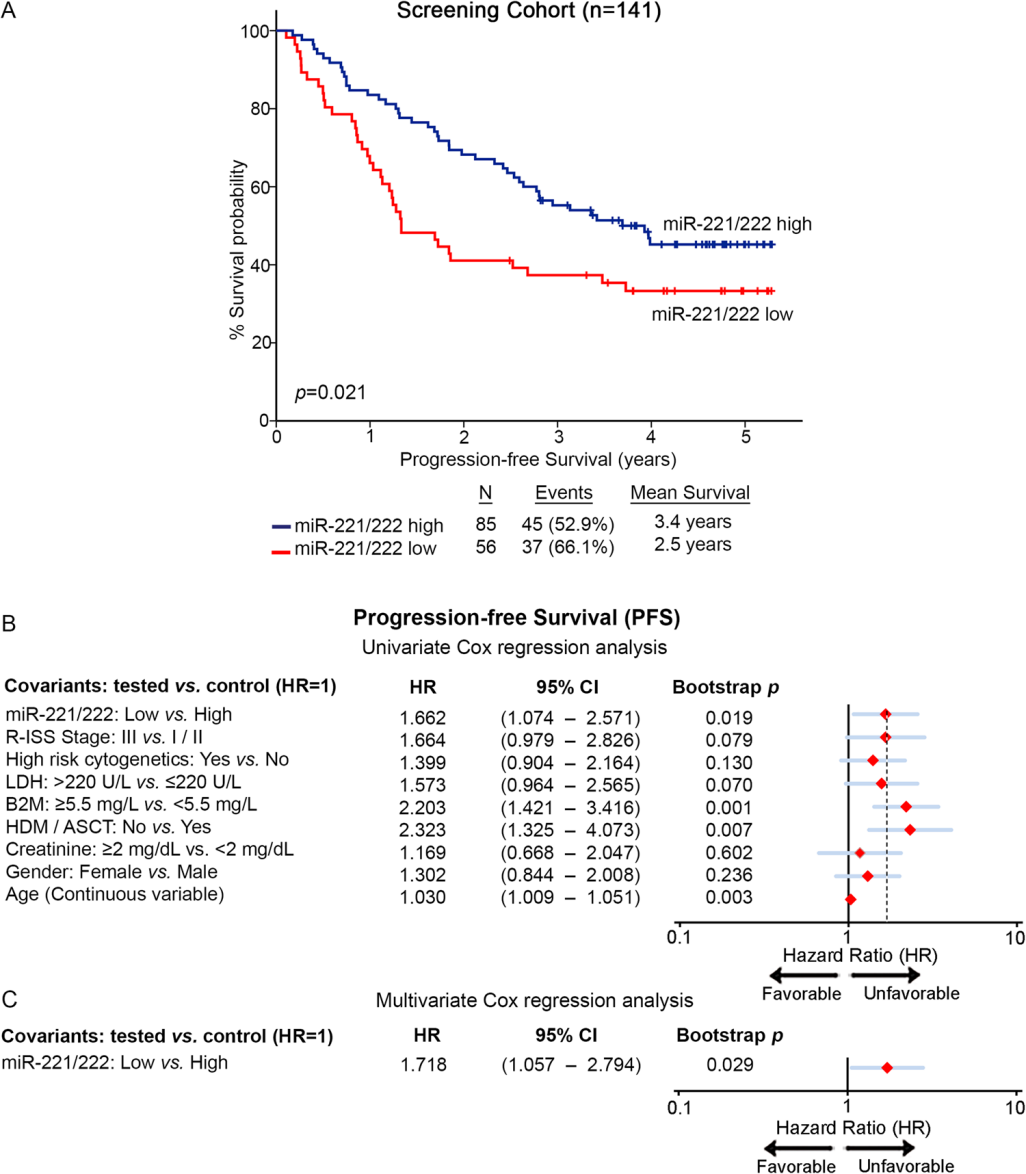
Patients with miR-221/222 loss are at significantly higher risk for short-term progression. **A** Kaplan–Meier survival curves for the progression-free survival (PFS) of the patients according to miR-221/222 levels in CD138+ plasma cells. *p* values calculated by log-rank test. **B**, **C** Forest plots of the univariate (**B**) and multivariate (**C**) Cox regression analysis for patients’ PFS. Multivariate analysis adjusted for miR-221/222 levels, R-ISS stage, high-risk cytogenetics, LDH, B2M, and creatinine levels, HDM/ASCT, gender, and age. Internal validation was performed by bootstrap Cox proportional regression analysis based on 1000 bootstrap samples. HR: Hazard Ratio; 95% CI: 95% confidence interval of the estimated HR.

The expression analysis of miR-221/222 in our screening cohort (n = 141) highlighted the strong correlation between miR-221 and miR-222 levels (Pearson test; r = 0.949, p < 0.001; Fig. 3A), unveiling their potent co-regulation in CD138+ plasma cells, allowing us to study the clinical value of miR-221/222 cluster in MM outcome (Figs. 3, 4). Besides R-ISS, miR-221/222 cluster loss was not associated with other clinicopathological covariates (Supplementary Fig. 2).

Furthermore, Kaplan—Meier and Cox regression analyses were assessed, utilizing patients’ death and disease progression (relapse and/or death; whichever came first) as clinical endpoints for the OS and PFS, respectively. miR-221/222 cluster expression was calculated utilizing the geometrical mean of miR-221 and miR-222 levels while using the X-tile algorithm, the 40^th^ percentile of miR-221/222 cluster levels was adopted as the optimal cut-off value classifying MM patients to “miR-221/222-high” and “miR-221/222-low” groups. Interestingly, the survival analysis of our screening cohort depicted the significantly worse survival (p = 0.002; Fig. 3B) and shorter PFS (p = 0.021; Fig. 4A) of patients presenting miR-221/222 loss. Moreover, univariate Cox regression analysis confirmed the shorter survival (HR: 2.382; 95%CI: 1.361–4.169; p = 0.002; Fig. 3E, Supplementary Table 2) and higher risk for disease progression (HR: 1.662; 95% CI: 1.074–2.571; p = 0.019; Fig. 4B, Supplementary Table 2) of the patients with loss of miR-221/222 in CD138+ plasma cells compared to those overexpressing the cluster. Intriguingly, as *MIR221/222* cluster is located on Xp11.3, we sought to investigate the potential impact of gender in miR-221/222 cluster expression and patients’ outcome (Supplementary Fig. 3). In this context, gender was not associated either with miR-221/222 levels (p = 0.472; Supplementary Fig. 3A) or with patients’ OS (p = 0.824; Supplementary Fig. 3B) and PFS (p = 0.232; Supplementary Fig. 3C). Additionally, multivariate Cox regression analysis clearly confirmed the ability of CD138+ miR-221/222 cluster loss to predict the worse survival outcome of the patients independently of their gender (Supplementary Fig. 3D, E).

Further strengthening our findings, the analysis of the two independent validation cohorts clearly confirmed the inferior outcome of the “miR-221/222-low” patients and the unfavorable prognostic value of miR-221/222 loss in MM. More precisely, the analysis clearly demonstrated the poor OS expectancy of the patients with loss of miR-221/222 cluster both in the Kryukov et al. (p = 0.007; Fig. 3C) and the Aass et al. (p = 0.005; Fig. 3D) cohort. Finally, to evaluate the independent prognostic value of miR-221/222 cluster in MM prognosis, multivariate Cox regression was performed. Indeed, the analysis highlighted miR-221/222 loss in CD138+ plasma cells as a potent predictor of worse survival (HR: 2.720; 95% CI: 1.464–5.052; p = 0.003; Fig. 3F, Supplementary Table 2) and higher risk for post-treatment progression (HR: 1.718; 95% CI: 1.057–2.794; p = 0.029; Fig. 4C, Supplementary Table 2) of MM patients, independently of R-ISS stage, high-risk cytogenetics [t(4;14), t(14;16), del(17p13), gain/amp(1q21)], B2M, LDH, creatinine levels, HDM/ASCT, patients’ gender and age.

### miR-221/222 cluster analysis significantly ameliorates the prognostic impact of the clinically established markers

Prompted by the independent prognostic utility of miR-221/222 cluster, we have investigated its ability to improve the value of the established and clinically used disease markers of a. response to 1st-line chemotherapy, b. high-risk cytogenetics [t(4;14), t(14;16), del(17p13), gain/amp(1q21)] and c. R-ISS stage.

**Fig. 5:**
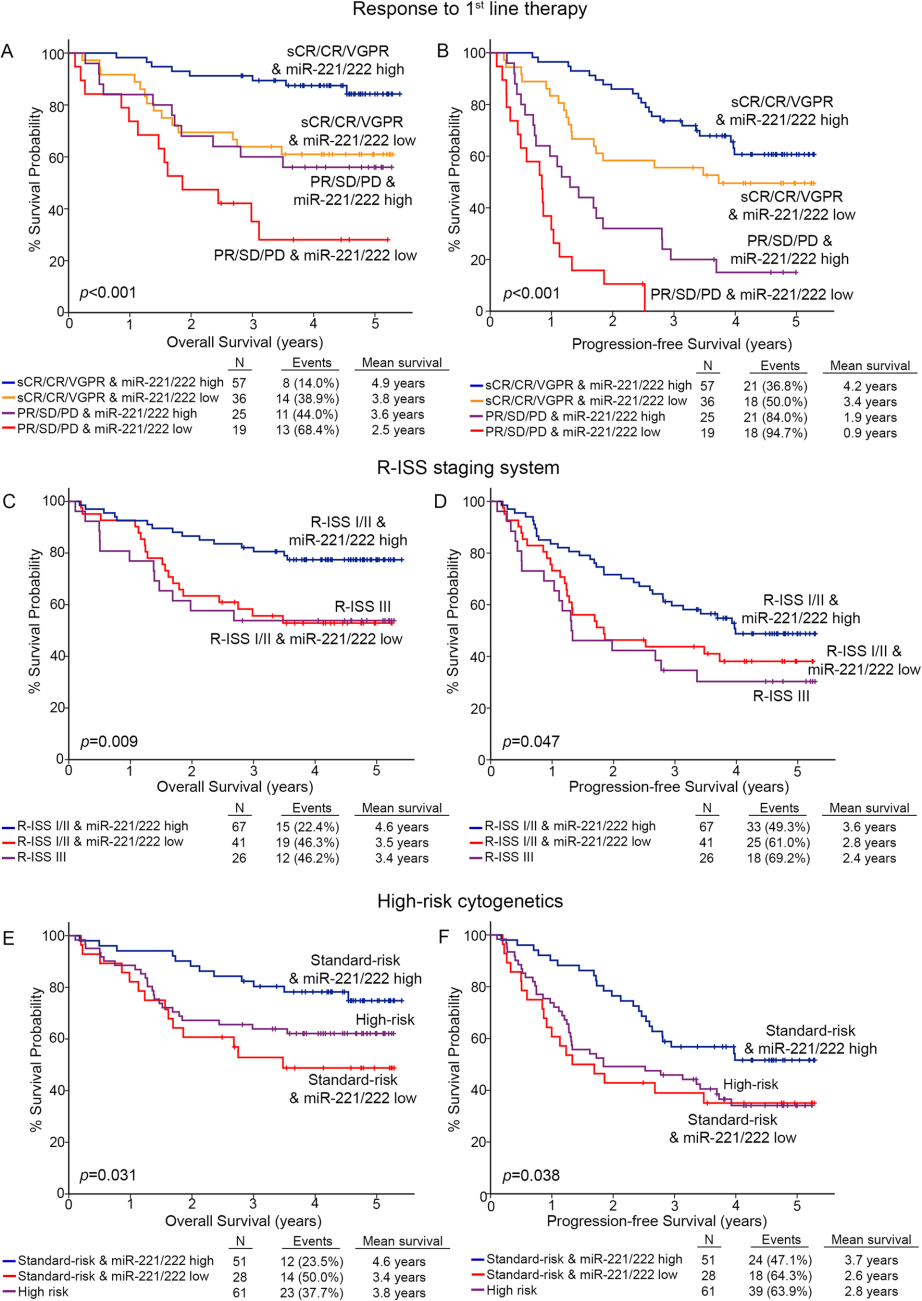
Evaluation of miR-221/222 levels increases risk-stratification efficacy and results in superior clinical benefit in MM prognosis. Kaplan–Meier survival curves for the overall survival (OS) and progression-free survival (PFS) of the patients according to miR-221/222 levels combined with response to 1st-line therapy (**A**, **B**), R-ISS stage (**C**, **D**), and high-risk cytogenetics (**E**, **F**). *p* values calculated by log-rank test. sCR stringent complete response, CR complete response, VGPR very good partial response, PR partial response, SD stable disease, PD progressive disease.

In this regard, the integration of miR-221/222 levels clearly improved patients’ risk-stratification and prediction of post-treatment outcome (Fig. 5). Focusing on response to 1st-chemotherapy, miR-221/222 loss could effectively predict patients with optimal treatment responses (stringent complete response-sCR, complete response-CR, and very good partial response-VGPR) with worse survival (p < 0.001, Fig. 5A) and at higher risk for disease progression (p < 0.001, Fig. 5B), compared to optimal responders overexpressing the cluster. Additionally, the combination of miR-221/222 levels with R-ISS stage resulted also to improved risk-stratification of the patients. More precisely, R-ISS I/II patients with miR-221/222 loss displayed significantly worse survival (p = 0.009, Fig. 5C), higher risk for short-term progression (p = 0.047, Fig. 5D) and prognosis analogous to R-ISS III group, compared to R-ISS I/II patients with higher miR-221/222 levels. Similarly, the analysis of miR-221/222 levels in CD138+ plasma cells could further stratify the standard-risk cytogenetics patients for disease prognosis. More specifically, standard-risk group with miR-221/222 loss showed significantly inferior disease outcomes in terms of OS (p = 0.031, Fig. 5E) and PFS (p = 0.038, Fig. 5F), resembling the high-risk group, compared to standard-risk patients with miR-221/222 overexpression.

**Fig. 6:**
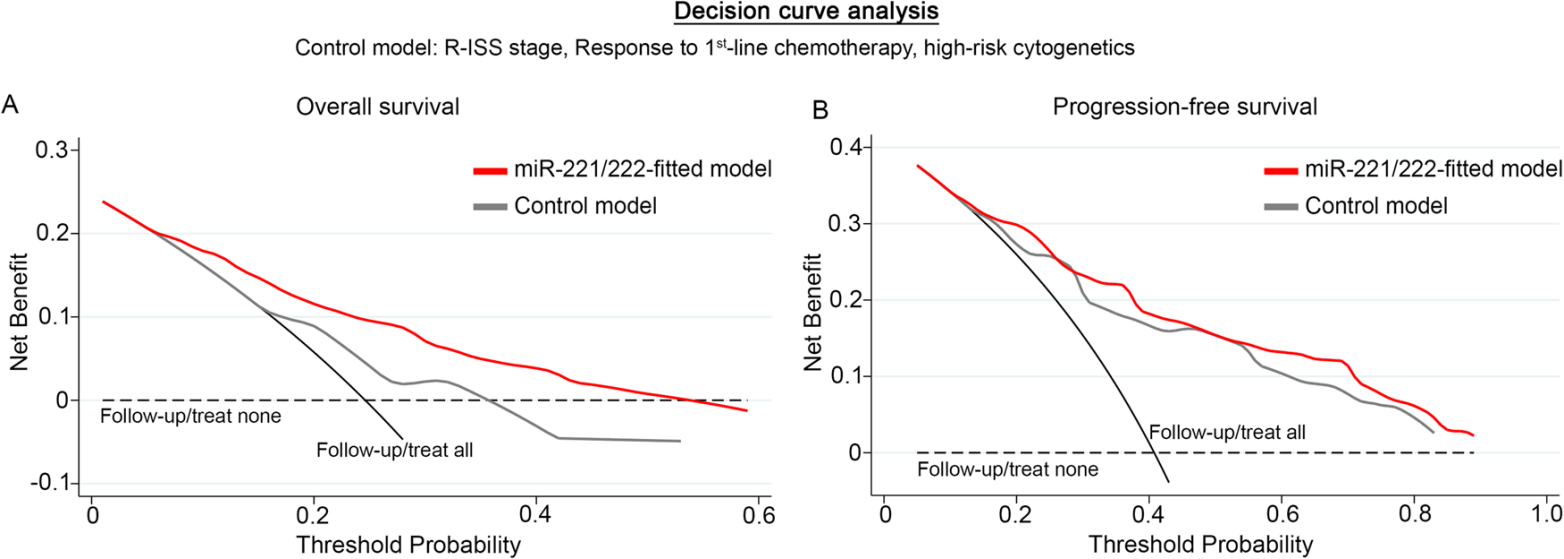
Decision curve analysis unveiled the improved clinical net benefit of the miR-221/222-fitted multivariate models. DCA curves of “miR-221/222-fitted” and “control” multivariate prediction models for the overall survival (OS; (**A**)) and progression-free survival (PFS; (**B**)) of the patients. Net benefit is plotted against various ranges of threshold probabilities. The control model consisted of R-ISS stage, response to 1st-line chemotherapy, and high-risk cytogenetics.

Finally, DCA was performed, according to Vickers et al., to study the clinical benefit of the multivariate prediction model incorporating miR-221/222 levels of CD138+ plasma cells along with the established and clinically used markers. The analysis demonstrated the significantly augmented clinical net benefit of the model integrating miR-221/222 loss in predicting patients OS (Fig. 6A) and PFS (Fig. 6B) compared to response to 1st-line therapy, R-ISS stage, and high-risk cytogenetics model. The superiority of miR-221/222-dependent prediction model, even at low threshold probabilities, is pivotal for the efficient risk-stratification and management of the MM patients.

## Discussion

Recent years have witnessed notable progress in the treatment of MM; however, the elevated relapse rates and therapy resistance continue to pose significant challenges in disease management. In this regard, the development of novel tools to improve patients’ risk-stratification and predict treatment outcome is urgently needed in translational clinical research [26]. Here, miRNA-seq was performed in CD138+ plasma cells of MM patients, aiming to unravel R-ISS-related miRNAs, underlining miR-221/222 as the most deregulated cluster in R-ISS III *vs*. R-ISS I/II patients.

Our study is the first addressing the clinical impact of miR-221/222 cluster in MM. The survival analysis of our MM screening cohort (n = 141) demonstrated the unfavorable prognostic value of miR-221/222 loss for disease outcome, as MM patients underexpressing miR-221/222 cluster in CD138+ plasma cells were at significantly higher risk for short-term relapse and worse survival following 1st-line chemotherapy. Furthermore, multivariate Cox regression models highlighted the poor treatment outcome of the MM patients with miR-221/222 loss, independently of the established disease markers and patients’ clinical data, including response to 1st-line therapy, R-ISS stage, high-risk cytogenetics, HDM/ASCT, B2M, LDH and creatinine levels, age and gender. In line with our findings, the analysis of Kryukov et al. 2016 (n = 149) and Aass et al. 2023 (n = 86) validation cohorts verified the worse survival outcome of the MM patients underexpressing miR-221/222 cluster, clearly confirming the unfavorable prognostic utility of miR-221/222 loss in MM. Finally, multivariate models incorporating miR-221/222 cluster expression with the clinically used prognostic markers of response to 1st-line therapy, R-ISS and high-risk cytogenetics resulted to significantly improved patients’ risk-stratification, leading to a superior MM prognosis model. More precisely, evaluation of miR-221/222 loss translated to advanced positive prediction of poor treatment outcome and disease progression within R-ISS I/II and standard-risk cytogenetics subgroups, as well as in both optimal and poor treatment responders.

The miR-221/222 gene cluster is located on Xp11.3, representing a widely studied cluster in numerous malignancies [27, 28]. miR-221/222 cluster exhibits a multifaceted role in the regulation of cancer cells’ behavior, in a context-specific manner. More precisely, miR-221/222 have been documented to regulate NF-κB and STAT3 signaling by directly binding to the *RelA* mRNA coding region, increasing its stability and stimulating colorectal cancer (CRC) development and progression [29]. Moreover, miR-221/222 have been highlighted to repress apoptotic mitochondrial pathway in glioblastoma by targeting PUMA [30]. On the hand, tumor suppression functions of the miR-221/222 cluster have been reported in hepatocellular carcinoma, in gastrointestinal stromal tumor pathogenesis and prostate cancer [31–33].

In MM, miR-221/222 inhibition was documented to stimulate autophagy and cell death, through ATG12 and p27^Kip1^ upregulation [34], as well as to trigger plasma cell apoptosis *via* PUMA overexpression [35, 36]. Focusing on cluster’s molecular function, antagomirs-mediated inhibition of miR-221/222 resulted in reduced cell proliferation through the upregulation of the p27^Kip1^ and p57^Kip2^ cyclin-dependent kinase inhibitors (CDKIs), while overexpression of the cluster triggered p27^Kip1^ downregulation and subsequently promoted S-phase, highlighting the potential impact of miR-221/222-p27^Kip1^/p57^Kip2^ axis in cell-cycle regulation of MM plasma cells [37].

At first glance, the documented here downregulation of miR-221/222 cluster in R-ISS III patients, as well as the association of miR-221/222 loss with worse treatment outcome of the patients contradicts the abovementioned functional role of miR-221/222 cluster in fostering the expression of p27^Kip1^/p57^Kip2^ CDKIs and reducing cell-cycle/proliferation of MM plasma cells. However, the prevalence of slow-cycling and mitotically quiescent cancer cells is strongly associated with chemotherapy resistance and post-treatment progression/relapse in multiple malignancies [38, 39]. Cancer quiescence reflects the ability of cancer cells to enter a reversible slow-cycling or mitotically dormant state upon tumor microenvironment stresses, such as (chemo)therapy, nutrient deprivation and immune surveillance, and represents a powerful self-protecting mechanism preventing cancer cell ‘damage’ [40]. In this regard, miR-221/222 inhibition has been highlighted to be essential for the maintenance of leukemic cell quiescence in adult acute and chronic lymphocytic leukemia [41, 42], while in vitro lentivirus-mediated restoration of miR-221/222 resulted in p27^Kip1^ targeting and cell-cycle progression, increased sensitivity to cytarabine and vincristine, and reduced relapse risk in adult acute lymphoblastic leukemia [43]. In that vein, the worse post-treatment outcome of the MM patients underexpressing miR-221/222 cluster could be explained by the active crosstalk of miR-221/222 with key mediators of cell-cycle and the promotion of MM plasma cells quiescence [42, 43]. Overall, miR-221/222 cluster loss could trigger CD138+ cells towards a slow-cycle and quiescent-like phenotype, providing survival advantage/resistance against 1st-line chemotherapy, and facilitating post-treatment disease relapse/progression.

Conclusively, miRNA-seq of CD138+ plasma cells highlighted the significant downregulation of miR-221/222 expression in R-ISS III MM patients, while the analysis of our screening and two independent validation MM cohorts confirmed the significantly higher risk for short-term progression and worse survival outcome of the MM patients with miR-221/222 loss. Additionally, multivariate regression models verified CD138+ lower miR-221/222 levels as an independent predictor of poor treatment and survival outcome of MM patients. Finally, evaluation of miR-221/222 expression with the clinically used disease markers of 1st-line chemotherapy, R-ISS stage and high-risk cytogenetics, resulted in enhanced risk-stratification specificity, superior positive prediction of patients’ poor treatment outcome and higher clinical benefit compared to the established markers alone, supporting miR-221/222 utility in modern diagnostics and management of MM patients.

## Supplementary information

Supplementary Table 1

Supplementary Table 2

Supplementary Figure 1

Supplementary Figure 2

Supplementary Figure 3

## Data Availability

All the data are available from the corresponding authors on reasonable request.
