# Supplementary material for: Refining precision prognostics in multiple myeloma: loss of miR-221/222 cluster in CD138+ plasma cells results in short-term progression and worse treatment outcome
Source: Blood Cancer J. 2025 Mar 15;15(1):41. doi: 10.1038/s41408-025-01248-2 (PMC11910569; doi:10.1038/s41408-025-01248-2)
Supplement: Supplementary file 1 — Supplementary Table 1 [file 41408_2025_1248_MOESM1_ESM.docx]

**Supplementary Table 1.** Deregulated miRNAs in R-ISS III *vs* R-ISS I/II among the top 30% abundant miRNAs in small RNA-seq.

| **R-ISS III  vs R-ISS I/II** | **miRNA** | **miRBase accession** | **Location** | **FC** | **Log2FC** |
| --- | --- | --- | --- | --- | --- |
| **Up** | hsa-miR-125b-5p | MIMAT0000423 | 19q13.41 | 2.641 | 1.401 |
|  | hsa-miR-150-5p | MIMAT0000451 | 19q.13.31 | 2.566 | 1.360 |
|  | hsa-miR-21-5p | MIMAT0000076 | 17q23.1 | 2.292 | 1.197 |
|  | hsa-miR-182-5p | MIMAT0000259 | 7q32.2 | 1.982 | 0.987 |
|  | hsa-miR-152-3p | MIMAT0000438 | 17q21.32 | 1.962 | 0.973 |
|  | hsa-miR-361-3p | MIMAT0004682 | Xq21.2 | 1.618 | 0.694 |
|  | hsa-miR-30a-5p | MIMAT0000087 | 6q13 | 1.397 | 0.483 |
|  | hsa-miR-142-3p | MIMAT0000434 | 17q22 | 1.360 | 0.443 |
|  | hsa-miR-191-5p | MIMAT0000440 | 3p21.31 | 1.344 | 0.427 |
|  | hsa-miR-15b-5p  hsa-miR-26a-5p  hsa-miR-26b-5p  hsa-miR-148b-3p  hsa-miR-374a-5p  hsa-miR-16-5p  hsa-miR-142-5p  hsa-miR-93-5p  hsa-miR-342-3p  hsa-miR-30d-5p  hsa-miR-7-5p  hsa-miR-30c-5p | MIMAT0000417  MIMAT0000082  MIMAT0000083  MIMAT0000759  MIMAT0000727  MIMAT0000069  MIMAT0000433  MIMAT0000093  MIMAT0000753  MIMAT0000245  MIMAT0000252  MIMAT0000244 | 3q25.33  3p22.2  2q35  12q13.13  Xq13.2  19q14.2  17q22  7q22.1  14q32.2  8q24.22  9q21.32  1p34.2 | 1.335  1.291  1.235  1.206  1.176  1.145  1.128  1.056  1.052  1.049  1.015  1.006 | 0.417  0.369  0.304  0.271  0.234  0.195  0.173  0.078  0.073  0.068  0.022  0.001 |
| **Down** | hsa-miR-148a-3p  hsa-let-7f-5p  hsa-miR-29b-3p  hsa-let-7i-5p  hsa-miR-29a-3p  hsa-miR-186-5p  hsa-miR-23a-3p  hsa-miR-25-3p  hsa-miR-27a-3p  hsa-let-7g-5p  hsa-miR-146a-5p  hsa-let-7d-5p  hsa-let-7a-5p  hsa-miR-30e-5p  hsa-miR-24-3p  hsa-miR-101-3p  hsa-miR-29c-3p  hsa-miR-103a-3p  hsa-let-7b-5p  hsa-miR-155-5p  hsa-miR-107  hsa-miR-34a-5p  hsa-miR-138-5p  hsa-miR-19b-3p  hsa-miR-20a-5p  hsa-miR-140-3p  hsa-miR-223-3p  hsa-miR-222-3p  hsa-miR-221-3p | MIMAT0000243  MIMAT0000067  MIMAT0000100  MIMAT0000415  MIMAT0000086  MIMAT0000456  MIMAT0000078  MIMAT0000081  MIMAT0000084  MIMAT0000414  MIMAT0000449  MIMAT0000065  MIMAT0000062  MIMAT0000692  MIMAT0000080  MIMAT0000099  MIMAT0000681  MIMAT0000101  MIMAT0000063  MIMAT0000646  MIMAT0000104  MIMAT0000255  MIMAT0000430  MIMAT0000074  MIMAT0000075  MIMAT0004597  MIMAT0000280  MIMAT0000279  MIMAT0000278 | 7p15.2  9q22.32  7q32.3  12q14.1  7q32.3  1p31.1  19p13.12  7q22.1  19p13.12  3p21.2  5q33.3  9q22.32  9q22.32  1p34.2  9q22.32  1p31.3  1q32.2  5q34  22q13.31  21q21.3  10q23.31  1p36.22  3p21.32  13q31.3  13q31.3  16q22.1  Xq12  Xq11.3  Xq11.3 | 0.985  0.985  0.980  0.963  0.962  0.954  0.943  0.935  0.930  0.899  0.884  0.881  0.875  0.847  0.841  0.837  0.829  0.807  0.791  0.776  0.739  0.703  0.607  0.583  0.540  0.432  0.360  0.353  0.332 | -0.022  -0.022  -0.029  -0.055  -0.056  -0.069  -0.084  -0.097  -0.105  -0.154  -0.177  -0.182  -0.193  -0.239  -0.250  -0.258  -0.270  -0.311  -0.338  -0.366  -0.436  -0.508  -0.720  -0.780  -0.889  -1.212  -1.473  -1.502  -1.591 |

FC: fold change
