# Supplementary material for: Refining precision prognostics in multiple myeloma: loss of miR-221/222 cluster in CD138+ plasma cells results in short-term progression and worse treatment outcome
Source: Blood Cancer J. 2025 Mar 15;15(1):41. doi: 10.1038/s41408-025-01248-2 (PMC11910569; doi:10.1038/s41408-025-01248-2)
Supplement: Supplementary file 2 — Supplementary Table 2 [file 41408_2025_1248_MOESM2_ESM.docx]

**Supplementary Table 2.** Cox regression analysis for the patients’ overall survival (OS) and progression-free survival (PFS) based on CD138+ miR-221/222 levels

|  | ***Univariate analysis*** | | | | | | | | | |
| --- | --- | --- | --- | --- | --- | --- | --- | --- | --- | --- |
|  | **Overall survival (OS)** | | | | | **Progression-free survival (PFS)** | | | | |
| **Covariant** | **HR^a^** | **95% CI^b^** | ***p*-value^c^** | **Bootstrap**  **BCa 95% CI^d^** | **Bootstrap**  ***p*-value^c^** | **HR^a^** | **95% CI^b^** | ***p*-value^c^** | **Bootstrap**  **BCa 95% CI^d^** | **Bootstrap**  ***p*-value^c^** |
| **CD138+ miR-221/222**  High expression  Low expression | 1.00  2.382 | 1.361-4.169 | 0.002 | 1.366-4.283 | 0.002 | 1.00  1.662 | 1.074-2.571 | 0.023 | 1.095-2.636 | 0.019 |
| **R-ISS Stage**  R-ISS I / II  R-ISS III | 1.00  1.760 | 0.911-3.401 | 0.092 | 0.808-3.453 | 0.107 | 1.00  1.664 | 0.979-2.826 | 0.060 | 0.928-2.959 | 0.079 |
| **High risk Cytogenetics**  No  Yes | 1.00  1.245 | 0.710-2.182 | 0.445 | 0.688-2.244 | 0.438 | 1.00  1.399 | 0.904-2.164 | 0.132 | 0.923-2.245 | 0.130 |
| **LDH**  ≤220 U/L  >220 U/L | 1.00  1.369 | 0.727-2.577 | 0.331 | 0.619-2.572 | 0.334 | 1.00  1.573 | 0.964-2.565 | 0.070 | 0.936-2.623 | 0.070 |
| **B2M**  <5.5 mg/L  ≥5.5 mg/L | 1.00  2.615 | 1.477-4.628 | 0.001 | 1.542-4.771 | 0.002 | 1.00  2.203 | 1.421-3.416 | <0.001 | 1.422-3.411 | 0.001 |
| **HDM/ASCT**  Yes  No | 1.00  3.366 | 1.433-7.904 | 0.005 | 1.526-12.88 | 0.004 | 1.00  2.323 | 1.325-4.073 | 0.003 | 1.333-4.248 | 0.007 |
| **Creatinine**  <2 mg/dL  ≥2 mg/dL | 1.00  1.706 | 0.891-3.266 | 0.107 | 0.783-3.136 | 0.099 | 1.00  1.169 | 0.668-2.047 | 0.585 | 0.611-1.985 | 0.602 |
| **Gender**  Male  Female | 1.00  0.938 | 0.535-1.646 | 0.824 | 0.529-1.634 | 0.817 | 1.00  1.302 | 0.844-2.008 | 0.233 | 0.856-2.058 | 0.236 |
| **Age** (continuous) | 1.053 | 1.025-1.082 | <0.001 | 1.029-1.084 | 0.001 | 1.030 | 1.009-1.051 | 0.005 | 1.009-1.052 | 0.003 |
|  | ***Multivariate analysis^e^*** | | | | | | | | | |
|  | **Overall survival (OS)** | | | | | **Progression-free survival (PFS)** | | | | |
| **Covariant** | **HR^a^** | **95% CI^b^** | ***p*-value^c^** | **Bootstrap**  **BCa 95% CI^d^** | **Bootstrap**  ***p*-value^c^** | **HR^a^** | **95% CI^b^** | ***p*-value^c^** | **Bootstrap**  **BCa 95% CI^d^** | **Bootstrap**  ***p*-value^c^** |
| **CD138+ miR-221/222**  High expression  Low expression | 1.00  2.720 | 1.464-5.052 | 0.002 | 1.412-6.033 | 0.003 | 1.00  1.718 | 1.057-2.794 | 0.029 | 1.023 -3.045 | 0.029 |
| **R-ISS Stage**  R-ISS I / II  R-ISS III | 1.00  0.781 | 0.280-2.177 | 0.637 | 0.195-3.613 | 0.743 | 1.00  0.755 | 0.340-1.676 | 0.490 | 0.264-2.462 | 0.580 |
| **High risk Cytogenetics**  No  Yes | 1.00  1.359 | 0.734-2.518 | 0.330 | 0.672- 3.199 | 0.372 | 1.00  1.532 | 0.939-2.500 | 0.088 | 0.919-2.819 | 0.101 |
| **LDH**  ≤220 U/L  >220 U/L | 1.00  1.494 | 0.589-3.791 | 0.398 | 0.327-4.752 | 0.503 | 1.00  1.837 | 0.956-3.528 | 0.068 | 0.747-3.970 | 0.115 |
| **B2M**  <5.5 mg/L  ≥5.5 mg/L | 1.00  2.150 | 0.969-4.769 | 0.060 | 0.831-6.165 | 0.095 | 1.00  2.475 | 1.323-4.631 | 0.005 | 1.320-6.143 | 0.014 |
| **Creatinine**  <2 mg/dL  ≥2 mg/dL | 1.00  0.792 | 0.362-1.734 | 0.792 | 0.280-2.242 | 0.606 | 1.00  0.551 | 0.278-1.095 | 0.089 | 0.222-1.163 | 0.116 |
| **HDM/ASCT**  Yes  No | 1.00  1.387 | 0.445-4.325 | 0.573 | 0.399- 6.137 | 0.625 | 1.00  1.747 | 0.798-3.825 | 0.163 | 0.729-4.959 | 0.215 |
| **Gender**  Male  Female | 1.00  0.988 | 0.540-1.809 | 0.988 | 0.485-2.109 | 0.979 | 1.00  1.342 | 0.841-2.141 | 0.218 | 0.799-2.427 | 0.258 |
| **Age** (Continuous) | 1.052 | 1.010-1.096 | 0.016 | 1.010-1.112 | 0.015 | 1.019 | 0.988-1.051 | 0.235 | 0.982-1.057 | 0.289 |

a: Hazard Ratio, b: 95% confidence interval of the estimated HR, c: Bootstrap *p*-value is based on 1000 bootstrap samples d: Bootstrap bias-corrected and accelerated 95% CI of the estimated HR based on 1000 bootstrap samples, e: Multivariate analysis adjusted for CD138+ miR-221/222 levels, R-ISS, high-risk cytogenetics, B2M / LDH / creatinine levels, HDM/ASCT, gender and age.
