# Supplementary figures and images for: Refining precision prognostics in multiple myeloma: loss of miR-221/222 cluster in CD138+ plasma cells results in short-term progression and worse treatment outcome
Source: Blood Cancer J. 2025 Mar 15;15(1):41. doi: 10.1038/s41408-025-01248-2 (PMC11910569; doi:10.1038/s41408-025-01248-2)

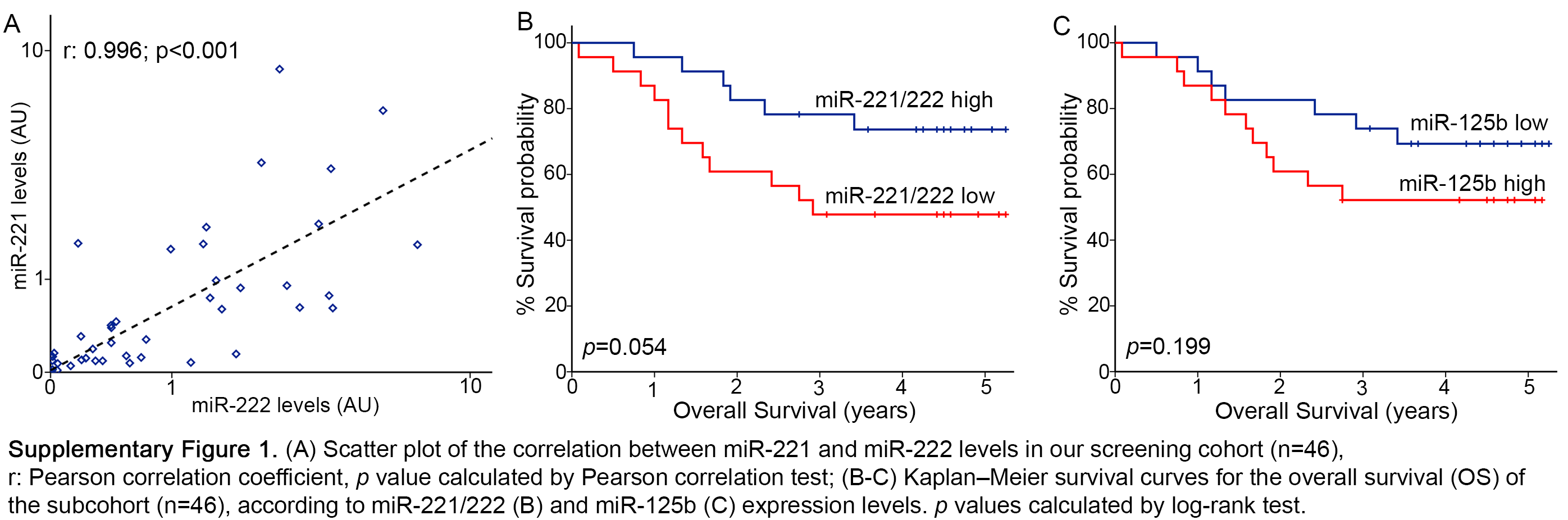

Supplement: Supplementary file 3 — Supplementary Figure 1 [file 41408_2025_1248_MOESM3_ESM.tif]

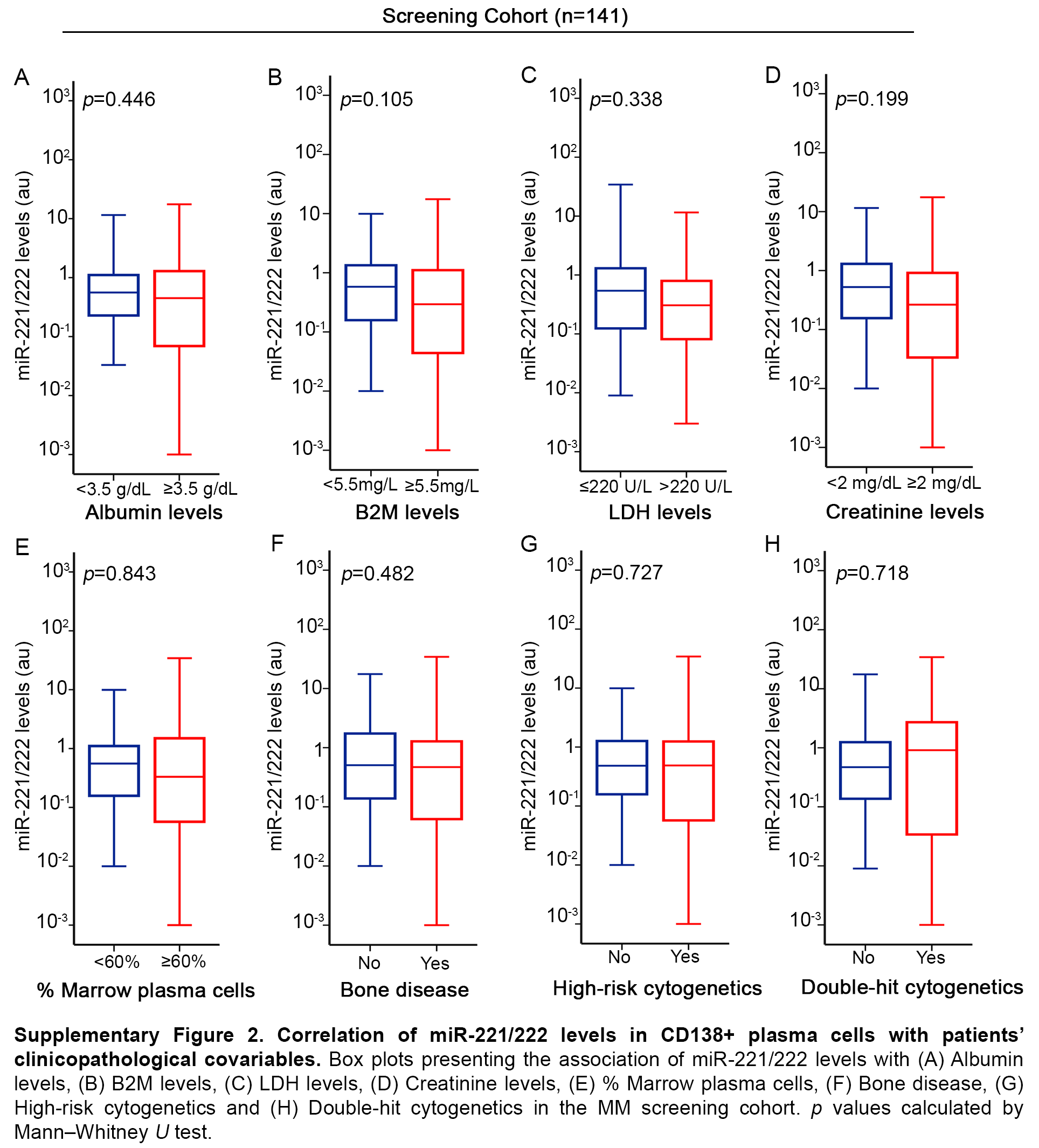

Supplement: Supplementary file 4 — Supplementary Figure 2 [file 41408_2025_1248_MOESM4_ESM.tif]

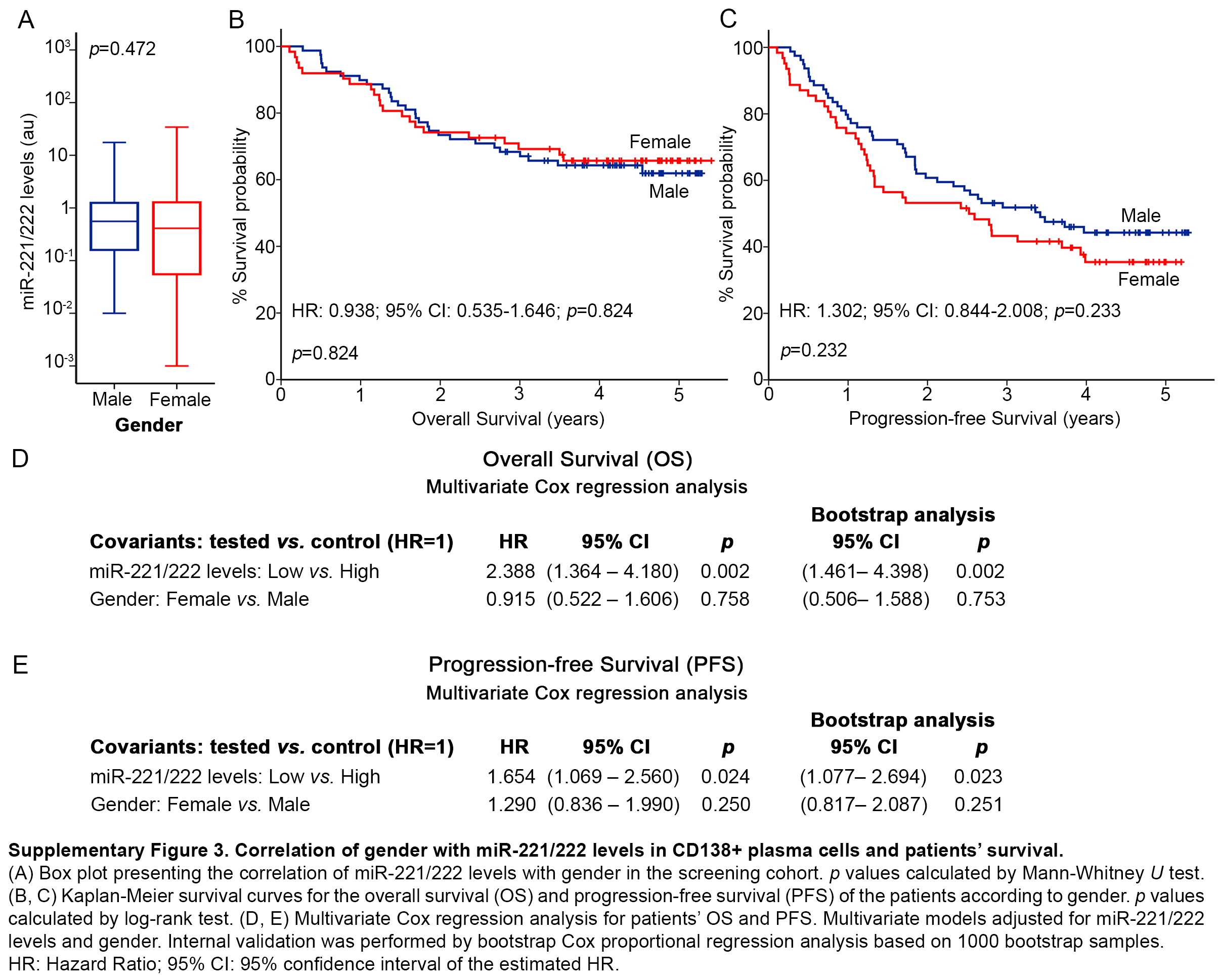

Supplement: Supplementary file 5 — Supplementary Figure 3 [file 41408_2025_1248_MOESM5_ESM.tif]
